# Neuroprotective Role of *N*-acetylcysteine against Learning Deficits and Altered Brain Neurotransmitters in Rat Pups Subjected to Prenatal Stress

**DOI:** 10.3390/brainsci8070120

**Published:** 2018-06-28

**Authors:** Liegelin Kavitha Bernhardt, K. Lakshminarayana Bairy, Sampath Madhyastha

**Affiliations:** 1 Department of Physiology, Melaka Manipal Medical College, Manipal Academy of Higher Education; Manipal 576104, India; kliz.1130@gmail.com; 2 Pharmacology, RAL College of Medical Sciences, Ras al-Khaimah Medical and Health Sciences University, Ras Al-Khaimah 11172, UAE; klbairy@yahoo.com; 3 Department of Anatomy, Faculty of Medicine, Kuwait University, Kuwait City 13060, Kuwait

**Keywords:** brain neurotransmitters, *N*-acetylcysteine, learning and memory, prenatal stress

## Abstract

Prenatal adversaries like stress are known to harm the progeny and oxidative stress, which is known to be one of the causative factors. *N*-acetyl cysteine (NAC), which is a potent antioxidant, has been shown to play a neuroprotective role in humans and experimental animals. This study examines the benefits of NAC on the prenatal stress-induced learning and memory deficits and alteration in brain neurotransmitter in rat pups. Pregnant dams were restrained (45 min; 3 times/day) during the early or late gestational period. Other groups received early or late gestational restrain stress combined with NAC treatment throughout the gestational period. At postnatal day (PND) 28, offspring were tested in a shuttle box for assessing learning and memory, which was followed by a brain neurotransmitter (dopamine, norepinephrine, and serotonin) estimation on PND 36. Late gestational stress resulted in learning deficits, the inability to retain the memory, and reduced brain dopamine content while not affecting norepinephrine and serotonin. NAC treatment in prenatally stressed rats reversed learning and memory deficits as well as brain dopamine content in offspring. These findings suggest that NAC protect the progeny from an undesirable cognitive sequel associated with prenatal stress.

## 1. Introduction

Stress is an integral part of day-to-day life and stress during pregnancy with its negative effects on neurobehavioral development in offspring is a subject of research demand. Even though many mechanisms underlying this neurobehavioral deficit have been identified, research minimizing or nullifying these adversaries are limited. Most of the literature in this regard are equivocal and they have proven beyond any doubt that stress during pregnancy (both humans and rodents) alter the normal hypothalamic-pituitary-adrenal axis, brain monoamines, and behavior in the progeny [1,2,3]. Among the possible causative factors, oxidative damage to the growing brain [4] results in cognitive impairment [5] along with changes in the levels of brain monoamines such as dopamine (DA), serotonin (5-HT), and noradrenaline (NE) [6], which is well established. In addition to affecting the brain monoamine system, prenatal stress induced oxidative damage in the growing brain also suppresses neuronal proliferation [7]. Such cellular damage in the developing brain could be attributed to overproduction of antioxidants. A balance between oxidants and antioxidants seems to play a critical role [8] during prenatal development. Using supplements of antioxidants has been an effective strategy for preventing stress-induced abnormality [8]. It is observed that chronic maternal stress induced a high cortisol level of the maternal blood, which entered the fetal brain and, thereby, altered fetal brain monoamine levels, which further cause personality changes [9]. There are many established results demonstrating neurobehavioral deficits due to altered brain amine levels in the growing rat brain. The study design in evaluating prenatal stress-induced neurobehavioral development most often utilized an open field, elevated plus a maze for anxiety-like behavior, forced a swim test for depression-like behavior and a Morris water maze for spatial learning. Very few studies utilized associative learning and memory models like the condition avoidance test, which is used in the present study.

In rodent models, it is evident that prenatal stress alters brain monoamines in offspring and this is linked to neurobehavioral deficits [10]. NE is one of the initial neurotransmitters found in the nervous system of the fetus and is believed to exert a trophic role in the development of the brain [11]. Decreased concentration of noradrenaline in the locus coeruleus and the cerebral cortex of rat offspring subjected to prenatal stress was observed in parallel with defensive freezing behavior [12]. Alteration in dopaminergic transmission and a decrease in dopamine transporter levels during early development have been shown to contribute to cognitive impairment [13,14,15]. Altered 5-HT expression during early postnatal development in rats who received prenatal stress [16,17,18] is involved in many behavioral activities including learning and memory especially since 5-HT is known to regulate neuronal development during the perinatal period.

Since NAC is a glutathione precursor, it is believed to be an ideal candidate that could prevent cognitive dysfunction among rat pups born from dams exposed to gestational stress. Our previous results clearly showed that NAC offers significant protection against oxidative damage in mothers exposed to late gestational stress by significantly improving the altered levels of serum MDA (Malondialdehyde), Glutathione Reductase, reduced glutathione, SOD (superoxide dismutase), and total antioxidants [18]. Several other studies have also demonstrated neuroprotective potentials of NAC in the developing brain [19,20,21,22]. Apart from these studies, few clinical studies have proven that NAC is favorable in bringing about neuroprotection in humans. Being able to pass through the placental and blood brain barriers, GSH levels increase, which was reported in experimental animal studies [23,24,25]. NAC can be considered a promising remedy that offers protection against the neurotoxic modifications brought about by prenatal stress. Our hypothesis is that antioxidant therapy could alleviate such deficits. We, therefore, used NAC, which is a well-known, potent antioxidant, to test its efficacy in minimizing the prenatal stress-induced neurobehavioral deficits and possible role of brain monoamines.

## 2. Materials and Methods

### 2.1. Animals

Female and male Albino Wistar rats (3–4 months of age), which weigh about 250 g, were obtained from an institutional animal house for the study. Day light cycle, temperature and humidity control were maintained. Animals were housed in polypropylene cages provided with paddy husk and were fed with standard food pellets and water. The experimental protocol was approved by the Institutional Animal Ethical committee. Rats were handled gently and all efforts were made to minimize their suffering and to limit the number of animals used.

Mating of rats: Female rats could mate with a fertile male rat (two female rats with one male rat) for four hours per day, after which, the female rats were confirmed for pregnancy. The presence of sperms in the vaginal smear was considered as positive for pregnancy and these rats were separated and allotted to separate groups (6 rats in each group) and gestational day 0 was designated. Pregnant rats were independently housed in separate cages and were monitored carefully for any pathological or abnormal changes throughout the pregnancy. After delivery, two healthy pups from each litter were selected for behavioral studies and for estimating brain neurotransmitter levels to avoid litter effects, which is appropriate for prenatal studies [26].

### 2.2. Animal Groups

Group 1. (Control (CON)) Pups of dams who received normal saline intra-peritoneally in a dose of 10 mL/kg body weight during the entire course of pregnancy.
Group 2. (NAC) Pups of dams who received NAC alone during the entire course of pregnancy.
Group 3. (EGS) Pups of dams subjected to restrain stress from day 1 to 10 of pregnancy.
Group 4. (LGS) Pups of dams subjected to restrain stress from day 11 of pregnancy until delivery.
Group 5. (EGS + NAC) Pups of dams subjected to restrain stress from day 1 to 10 of pregnancy along with NAC treatment throughout pregnancy.
Group 6. (LGS + NAC) Pups of dams subjected to restrain stress from day 11 of pregnancy until delivery along with the NAC treatment throughout pregnancy.

All pregnant rats delivered at around the 21st to 23rd day of gestation. Pups of all groups were raised with their biological mothers until 21 days after birth and were then weaned.

### 2.3. Stressing Procedure

Wire mesh restrainers were made to use for subjecting the pregnant dams to restrain the stress procedure. This procedure was regularly performed for 45 min three times per day. The restrainer was made of a wooden base. A stainless-steel wire mesh hinged to the base and a pad padlock with clasp. Restrainers with two different dimensions were prepared including one for stressing rats during early pregnancy (11 cm (L) × 6cm (B) × 6 cm (H)) and the other for stressing rats during late pregnancy (11 cm (L) × 8 cm (B) × 8 cm (H)). Immobilization in a restrainer is considered one of the best-known models of stress since it represents emotional as well as physical aspects of stress [27].

### 2.4. Chemicals

Chemicals and reagents used were of an HPLC or analytical grade (Sigma, St. Louis, MO, USA). *N*-acetyl cysteine was purchased from Lobo chemicals and procured locally from Sri Durga laboratories, Mangalore.

*N-acetylcysteine treatment:* The acute oral toxicity of *N*-acetyl cysteine is low e.g., LD 50 > 10,000 mg/kg body weight and all NAC-related effects observed were marginal. The optimal favorable results were obtained from a dose ranging from 0.6 g/day in the human clinical trial [28]. Applying this dose to the rat model and from values of previous studies in rats such as by Basyigit et al. [29]. A dosage of 10 mg/kg body weight NAC dissolved in physiological saline was selected for this study.

### 2.5. Neonatal Study Parameters

The pups were observed daily from the day of birth until weaning for neonatal and developmental parameters. Gestational length and mortality rate were screened to assess the safety of NAC administration during pregnancy.

### 2.6. Learning and Memory Test: Condition Avoidance Test (Shuttle Box Test)

This test is employed to evaluate associative learning and memory. In this study, the ability of the rat to evade an aversive experience by learning to accomplish a specific behavior in response to a stimulus signal can be assessed. The shuttle box is made of a closed wooden box with shutters in the front. The floor area consists of grids separated into two portions by a median grid. The two parts are connected to separate electric circuits. The box consists of a buzzer inside it. Rats were placed in the box and allowed to explore for 5 min. 10 s after this, a discriminative stimulus was provided through the buzzer during which the rat could escape the shock by moving to the other compartment. If the rat failed to escape the discriminative stimulus, a shock of 2.5 mA was delivered for a maximum period of 10 seconds during which it could cross to the other side to escape the shock. The contingency for avoiding the shock is a single crossing over the median grid from one side of the shuttle box to the other. The test consisted of 30 trials daily for 5 consecutive days. The number of shock avoidances increased from day 1 to day 5 of the test under normal circumstances. The average shock avoidance numbers on all 5 days were called the mean score during the 5 days of testing. Any decrease in this score is an indication of a learning impairment. Each rat was retested one week after the last trial to assess retention of memory. A comparison of the rat’s performance with its previous performance provides a memory assessment and is presented as the percentage of the retention score (RTS), which is calculated based on the methodology in Sampath Madhyastha et al. (2002) [30]. Offspring were assessed on day 28 of postnatal life.

RTS = Mean of retest score × Mean scoring during 5 days of testing ÷ Mean score during day 5 of testing

Any decrease in the retest score and retention score is an indication of memory impairment. Each rat is trained for 4 days before starting the test.

### 2.7. Sacrifice and Tissue Processing for Estimating Whole Brain Neurotransmitter Levels

Thirty-six days old pups were sacrificed by decapitation. Brains were rapidly removed, placed on an ice plate, and rinsed with cold normal saline. Tissues were then weighed and stored in a deep freezer (−20 °C) until neurotransmitter assays were carried out. For determining NE, DA, and 5-HT levels, tissues were homogenized in 2 mL of 0.1 M PCA and then the homogenate was centrifuged by a cold centrifuge at 5000 g for 20 min The resultant supernatant was separated. Quantitative determination of whole brain neurotransmitters was executed using a commercially available competitive ELISA kit (kit Dopamine ELISA, Noradrenalin ELISA, IBL-Hamburg and Serotonin ELISA, LDN, Nordhorn). The procedure consisted of two phases—extraction and quantification—performed following the manufacturer’s instructions [31].

### 2.8. Statistical Analysis

All results represent a mean ± S.E.M. The significance of differences among the groups was assessed using the one-way analysis of Variance (ANOVA) test followed by Tukey-Kramer Multiple Comparisons Test. *p* values < 0.05 were considered significant. Analysis were made using SPSS software version 16 (IBM, Chicago, IL, USA).

## 3. Results

The general health condition of pregnant rats when stressed or during the NAC treatment period and the health of the pups were monitored carefully. Body weights were noted at frequent intervals to ensure that the results were not affected by any other illness. No such effects were observed among the rats tested.

### 3.1. Effect of Prenatal Stress and NAC on Neonatal Parameters

No significant difference in gestational length, litter size, day of upper teeth eruption, and day of eye opening was observed between any of the groups (Table 1).

There was no significant difference in the mortality rate between any of the groups (Table 2).

### 3.2. Effect of Prenatal Stress and NAC on Learning and Memory (PND 28)

**Figure 1:**
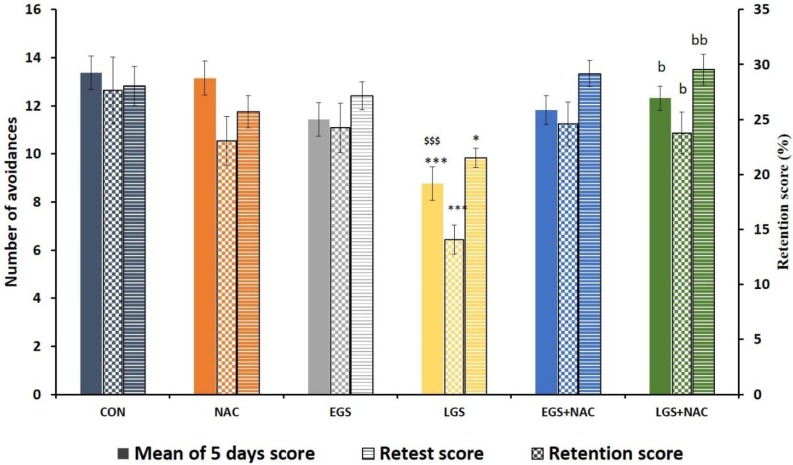
Effect of early and late gestational stress and NAC treatment on avoidance activities by 28-day-old rats in the shuttle box test. One-way ANOVA, Tukey-Kramer Multiple Comparisons Test, each bar represents mean ± SEM, *n* = 12), CON vs. LGS, * *p* < 0.05, *** *p* < 0.001; LGS vs. LGS + NAC ^b^ *p* < 0.05, ^bb^ *p* < 0.01, NAC vs. LGS, ^$$$^ *p* < 0.001.

Offspring who received late gestational stress (LGS) showed a significant decrease in the mean score during 5 days of testing (*p* < 0.001), a significant decrease in the retest score (*p* < 0.05), and a significant decrease in the retention score (*p* < 0.001) when compared with the control offspring. Rats who received late gestational stress and NAC treatment showed a significant increase in the mean score during 5 days of testing (*p* < 0.05). The same was found in the retest score (*p* < 0.01) and the retention score (*p* < 0.05) when compared to pups who received only late gestational stress. These effects were observed only in the LGS group but not in the EGS rats (Figure 1). This result demonstrates that prenatal NAC treatment reverses/minimizes stress-induced learning and memory disabilities in stressed offspring.

### 3.3. Effect of Prenatal Stress and NAC on Whole Brain Dopamine Levels

**Figure 2:**
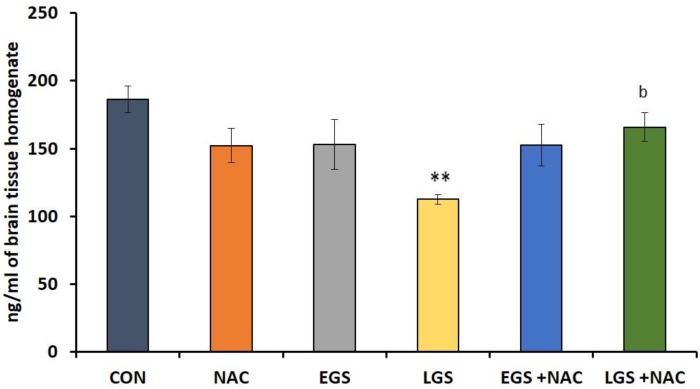
Effect of prenatal stress and NAC on whole brain homogenate dopamine levels (nmol/mL) at PND 36 in rat offspring. One-way ANOVA, Tukey-Kramer Multiple Comparisons test, each bar represents mean ± SEM, *n* = 6. Data are expressed as mean ± SEM. CON vs. LGS, ** *p* < 0.01, LGS vs. LGS + NAC ^b^ *p* < 0.05.

A significant decrease (*p* < 0.01) in the dopamine level was observed in the whole brain homogenate of offspring exposed to late gestational stress in comparison with the control. However, this type of alteration was not observed in offspring exposed to early gestational stress. NAC treatment in the late gestational stress group has reversed this effect by significantly elevating (*p* < 0.05) brain dopamine level (Figure 2).

### 3.4. Effect of Prenatal Stress and NAC on Brain Norepinephrine Levels

**Figure 3:**
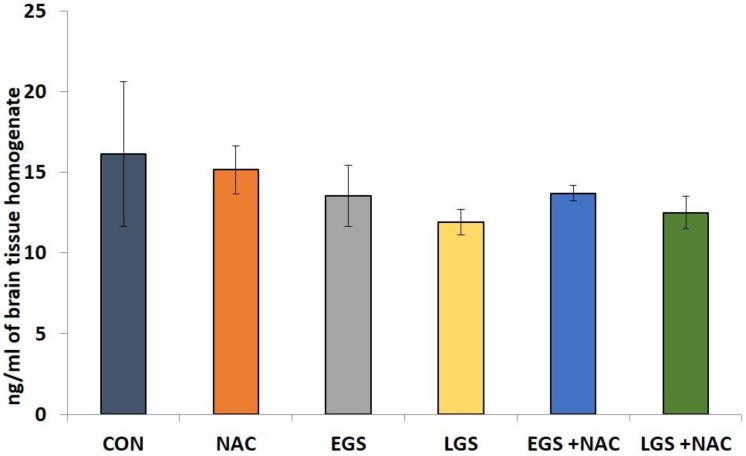
Effect of prenatal stress and NAC on whole brain homogenate norepinephrine levels (nmol/ml) at PND 36 in rat offspring. One-way ANOVA, Tukey-Kramer Multiple Comparisons Test, each bar represents mean ± SEM, *n* = 12. Data are expressed as mean ± SEM.

No significant differences (*p* > 0.05) in brain NA levels was observed between any of the groups (Figure 3).

### 3.5. Effect of Prenatal Stress and NAC on Brain Serotonin (5HT) Levels

**Figure 4:**
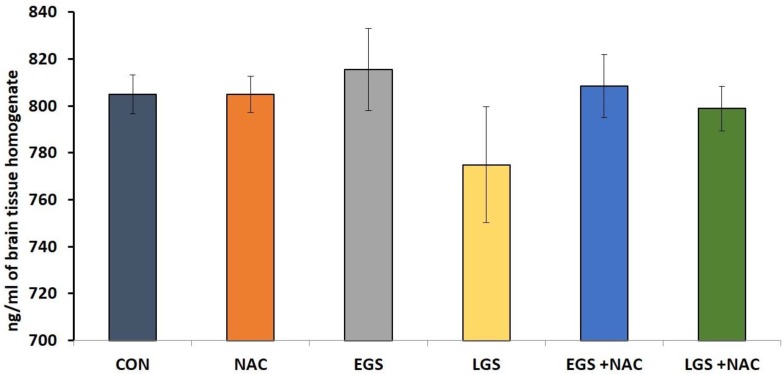
Effect of prenatal stress and NAC on whole brain homogenate serotonin levels (nmol/mL) at PND 36 in rat offspring. One-way ANOVA, Tukey-Kramer Multiple Comparisons Test, each bar represents mean ± SEM, *n* = 12. Data are expressed as mean ± SEM.

There was no statistically significant variation in the level of brain 5HT between any of the groups (Figure 4).

## 4. Discussion

Stress during pregnancy is known to have a deleterious effect on offspring especially in terms of neurobehavioral development involving brain monoamines. The oxidative stress in the developing brain is one of the causative factors. In this experiment, we examine the effect of antioxidant therapy during gestation on learning, memory, and brain monoamines.

In teratogenic study models, information regarding gestational length, birth weight, litter size, eruption of incisor teeth, and day of eye opening would provide significant information. In our study, stress during different periods of gestations has not shown any adverse effects in these parameters. Kvarik et al. [32] in their study, while addressing maternal stress during different periods of pregnancy, did not observe any delay in eye or ear opening. Equivalent results were reported by earlier studies where these parameters were included [33,34]. Early or late gestational stress with or without NAC treatment does not exert a profound influence on these initial physical land marks and mortality.

Studies with regards to gestational stress-induced neurobehavioral deficits in offspring is an established fact. However, there are differences in results based on timing of neurobehavioral assessment [32] postnatally, gender of the animals [35,36], and selections of the assessment models in offspring [37,38,39]. Few studies have focused on finding out whether these neurobehavioral deficits are associated with timing of stress during pregnancy. In our study, early gestational stress has not affected the learning and memory outcome when measured at postnatal day 29, but offspring subjected to late gestational stress showed a learning disability and memory impairment in terms of task avoidance (Figure 1). There are many similar findings in human models as well as rodent models [40,41,42] except for a study by Huizink [43] where early gestational stress resulted in problematic infant behavior. The learning and memory deficits observed on postnatal day 29, which corresponds to 11 years of age to 16 years of age in a human [44], show a significant period where learning and memory play a vital role in designing the future life quality of an individual. Even though the third and fourth week of intrauterine life is most susceptible to teratogens that cause gross and severe form of birth defects, late gestational events also produce neurobehavioral deficits. Therefore, protection during this period of gestation needs ample attention, which requires understanding of probable mechanisms involved during late stages of gestation in inducing neurobehavioral deficits. An increased level of circulating glucocorticoids and its extensive impact on the developing brain [45] as well as altered expression of brain derived neurotrophic factor and brain monoamines [7], the possible involvement of oxidants and the relation to glucocorticoid hormones in stress have been proposed [46]. An enhanced glucocorticoid effect can compromise mitochondrial respiration and, thereby, contribute to ROS production. This could lead to neuronal damage and could be associated with neurobehavioral deficits in offspring. Therefore, preventing overproduction of oxidants by using antioxidants is expected to minimize or prevent the neurobehavioral deficits.

Oxidative stress produced by prenatal stress has resulted in neuronal loss in offspring [18]. This could involve brain monoamines in developing offspring brain. Prenatal NAC treatment enhanced the activity of the antioxidant defense system in the offspring who received prenatal restrain stress. In this study, NAC has reversed the oxidative damage in all the parameters on postnatal day 24 and brain MDA level and GSS-Rd level on postnatal day 48 [47]. In the present study, prenatal NAC treatment reversed the learning and cognitive dysfunctions in prenatally stressed rats. The mechanism by which NAC would have offered protection against behavioral toxicity is not completely clear. It is well-known that NAC is a potent antioxidant, provides cysteine, and replenishes GSH. These actions may be responsible for its protective role against prenatal stress induced behavioral toxicity. Human volunteers suffering from Alzheimer’s showed benefits from some but not all cognitive dysfunctions after being treated daily with 50 mg/kg/day for six-month duration [48]. Therefore, we provide experimental support for the hypothesis that NAC may play a pivotal role as an antioxidant in terms of neuroprotection against various neurotoxic conditions.

Even though the gestational stress-induced neurobehavioral alterations were linked to oxidative stress and brain monoamines in the developing brain, their relations were not adequately addressed. We found a statistically significant reduction in the whole brain dopamine level in offspring exposed to late gestational stress. However, levels of NE and 5-HT did not show any significant alteration. The formation of dopaminergic neurons begins at around gestational day 10.5 in rodents [49] and, in most areas of the brain, maturation of the dopaminergic system is considered to be complete and functionality is attained weeks after birth in rats [50] as well as in humans [51]. Presently, it is being hypothesized that reduced dopamine activity in the D1 receptor of the prefrontal cortex causes cognitive impairment and adverse behavioral patterns seen in schizophrenia cases [49]. Dopaminergic circuits are thought to be directly or indirectly targeted by stress and glucocorticoids [49]. Early life stress has shown not only to alter dopaminergic systems [52], but also to modify dopamine transporter, function, sensitivity, and DA-receptor expression [49].

Late gestational stress induced a fall in the level of dopamine parallels with behavioral deficits seen in response to the same period of stress. It can be noted here that a decreased level of dopamine may partly be responsible for the behavioral deficits. Early life stress has been linked to increased vulnerability of the offspring to anxiety and depression [53,54], which further seems to involve modification of the dopaminergic tone [49]. Baier and coworkers [55] reported that alteration in the level of neurotransmitter dopamine (DA) resulted in impaired fetal brain development and, therefore, increased vulnerability to adverse behavioral patterns due to prenatal stress in rodents. Alteration in the mid-brain dopamine system was observed in maternally stressed mice offspring. This is parallel with behavioral modification such as diminished adaptation to novel environment [56]. Significantly lower levels of dopamine were seen in the locus coeruleus of offspring subjected to gestational stress along with a defensive freezing-like behavior [12]. These studies point out that alteration in brain catecholamine levels induced by prenatal stress could cause various forms of behavioral deficits.

We did not find any significant alteration with respect to 5-HT concentration in whole brain samples of adolescent offspring (PND 36).

No change in NE was seen in the olfactory bulb of offspring of rats exposed to late gestational restrain stress on PND 7 [57]. Conflicting results with respect to prenatal stress induced neurotransmitter levels exist because the stress responses vary with the kind of stress, its severity, duration, and, importantly, the postnatal age at which the assessments were carried out. In this study, we did not obtain any significant alteration in the level of NE in the whole brain on PND 36. Therefore, by considering a decreased concentration of DA in the whole brain at PND 36, it can be assumed that the DA system is more vulnerable to prenatal stress compared to the other two brain monoamines estimated. Apart from the changes in the level of monoamines that serve as a possible cause for behavioral alterations, oxidative stress, and sympathetic stimulation leads to decreased uterine blood flow, involvement of Na+/K+-ATPase activity, and altered HPA axis have also shown to contribute in varied degrees to the neurotoxic outcomes of gestational stress [18]. This is the subject of active research.

In this study, NAC successfully enhanced the brain DA level, which was reduced in offspring who received late gestational stress. No studies have investigated the effect of NAC on gestational stress-induced modification of brain neurotransmitter levels to the best of our knowledge. Oxidative stress has been linked to dopaminergic degeneration [58] and the sustainability of dopaminergic neurons can be reduced by the generation of reactive oxygen species [49]. From its effective antioxidant capacity, NAC possibly caused protection against dopamine depletion in this study. Moreover, it has been proven that glutathione depletion, which is an early indicator of neurodegeneration, can promote DA neuronal cell death and reduction in dopamine levels [59]. NAC is able to promote glutathione synthesis [60], which has also been proven in our study [47]. This may prevent the damage to DA neurons brought about by prenatal stress. A possible role of NAC to replenish the glutathione store in the brain may provide an explanation of how NAC improved the dopamine level in stressed offspring in our study. Moreover, NAC has been found to bring about modification of altered dopamine release in rat striatal neurons following treatment with amphetamine [61]. The cognition-improving actions of amphetamine occurs through its indirect stimulation of the dopamine receptor D1 in the prefrontal cortex [62]. In monkeys treated with methamphetamine, intravenous NAC administration attenuates the fall in dopamine transporter expression [63]. Low concentrations of NAC enhance the release of the neuronal vesicular dopamine [61]. It is also known that glutathione can increase glutamate-evoked dopamine release [64].

## 5. Conclusions

In conclusion, our data suggest that late gestational stress induces fear-related memory impairment in offspring during the peri-adolescent period and results in a decreased brain dopamine level. However, no change in the levels of brain serotonin and noradrenalin were observed. Additionally, we have demonstrated that behavioral as well as neurochemical alterations are reversed by prenatal NAC treatment. Therefore, this study demonstrates that a decreased dopamine level may partly contribute to prenatal stress-induced behavioral toxicity. Based on the results of this report, we propose that prenatal treatment with NAC would be an effective strategy for preventing cognitive dysfunction associated with prenatal stress.

## Figures and Tables

**Table 1:** Neonatal parameters following early and late gestational stress and prenatal NAC treatment.

	CON	NAC	EGS	LGS	EGS + NAC	LGS + NAC
Gestational length (*n* = 12)	21.66 ± 0.14	21.41 ± 0.15	21.66 ± 0.14	21.5 ± 0.19	21.58 ± 0.14	21.5 ± 0.15
Litter size	9.66 ± 0.22	9.25 ± 0.65	8.5 ± 0.65	9.08 ± 0.65	9.66 ± 0.22	9.58 ± 0.22
Day of upper incisor eruption (*n* = 24)	7.95 ± 0.35	7.70 ± 0.55	8.29 ± 0.46	8.08 ± 0.82	7.95 ± 0.55	8.04 ± 0.55
Day of eye opening (*n* = 24)	8.20 ± 0.72	8.16 ± 0.13	8.41 ± 0.71	8.62 ± 0.57	8.12 ± 0.67	8.12 ± 0.74

Data are expressed as mean ± SEM. Animal groups: CON: control, NAC: pups received prenatal *N*-acetylcysteine during entire gestation period. EGS: pups exposed to prenatal stress during gestation day1 to 10. LGS: pups exposed to stress during gestation day 11 until delivery. EGS + NAC: pups received prenatal stress during gestation day 1 to 10 along with NAC for the entire gestation period. LGS + NAC: pups received prenatal stress during gestation day 11 until delivery along with NAC for the entire gestation period. (One-way ANOVA, Tukey-Kramer Multiple Comparisons Test).

**Table 2:** Effect of early or late gestational stress and prenatal NAC treatment on the mortality rate.

Animal Groups	Mean Number of Pups	Still Born Pups	Until Post-Natal Day 7	Post-Natal Day 7 to 21
**CON**	9.6	8.3%	0%	0%
**NAC**	9.2	16%	0%	0%
**EGS**	8.5	5.8%	0%	0%
**LGS**	9.08	5.5%	0%	0%
**EGS + NAC**	9.6	9.75%	0%	0%
**LGS + NAC**	9.6	6.09%	0%	0%

One-way ANOVA, Tukey-Kramer Multiple Comparisons Test. *p* > 0.05.
